# *Bifidobacterium animalis* subsp. *lactis* genome resources and metabolite profiling at the strain level and their ability to alleviate anxiety-like behavior in a sleep-deprived mouse model

**DOI:** 10.1016/j.engmic.2025.100228

**Published:** 2025-07-30

**Authors:** Yao-Kun Zhang, Liang Zhang, Xue Ni, Shu-Wen Zhang, Min-Zhi Jiang, Si-Lu Zhang, Guo-Xun Xiao, He Jiang, Ming-Xia Bi, Yu-Lin Wang, Chang Liu, Shuang-Jiang Liu

**Affiliations:** a State Key Laboratory of Microbial Technology, Shandong University, Qingdao 266237, China; b WONDERLAB Innovation Centre for Healthcare, Shenzhen Porshealth Bioengineering Co., Ltd, Guangdong 518000, China

**Keywords:** *Bifidobacterium animalis* subsp. *lactis*, Probiotic strains, Metabolite profiling, Sleep-deprived mouse model, Anxiety-like behavior

## Abstract

*Bifidobacterium animalis* subsp. *lactis* is a well-known probiotic with potential benefits for alleviating sub-health symptoms, including immune dysfunction and anxiety. Given the strain-specific nature of its probiotic effects, identifying effective strains for sub-health alleviation is crucial. In this study, we characterized 16 *B animalis* subsp. *lactis* isolates from fecal samples and probiotic sources. We assessed the genotype-phenotype correlations related to growth, carbon source utilization, and stress tolerance *in vitro*. Subsequently, we profiled 107 metabolites (including 28 alcohols and 17 esters) and quantified the levels of short-chain fatty acids and three other organic acids. Three *B. animalis* strains, GOLDGUT-BB21, WLBA7, and WLBA6, were selected and evaluated in a sleep-deprived mouse model. *In vivo*, WLBA3 reduced inflammation and oxidative stress by inhibiting the NLRP3 inflammasome pathway and modulating gut microbiota (e.g., *Lactobacillus* and *Alistipes*), which in turn significantly improved weight gain and fatigue resistance, attenuated cognitive function, and anxiety-like behavior. These findings provide insights into the diversity of *B. animalis* subsp. *lactis* strain resources and highlight the potential of WLBA3 as a candidate for alleviating sub-health symptoms.

## Graphical abstract

**Figure:**
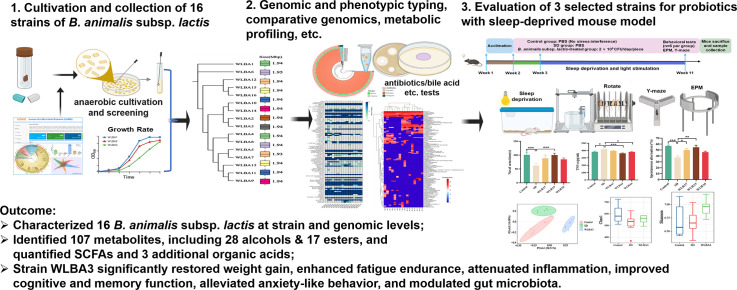

## 1 Introduction

Sub-health, an intermediate state between health and illness, is exacerbated by the demands of modern lifestyles and pervasive stress [1]. Manifestations of sub-health include persistent, non-specific symptoms, such as fatigue, gastrointestinal disturbances, compromised immunity, and mood disturbances (e.g., anxiety-like behavior) [[2], [3], [4], [5]]. Therefore, a comprehensive evaluation of sub-health requires a multifaceted assessment that encompasses general stress levels, as well as physical and psychological status [1]. Global surveys conducted by the World Health Organization indicate that approximately 75 % of the world’s population experiences sub-health problems [6], with rates exceeding 65 % in China [7]. Prolonged sub-health can predispose individuals to chronic diseases, including cardiovascular diseases, hypertension, stroke, diabetes, chronic obstructive pulmonary disease, and cancer [8]. Given the established link between sleep and overall health, unhealthy sleep patterns, such as reduced sleep duration and impaired sleep quality, are significant contributors to sub-health [9]. Consequently, sleep-deprived (SD) mouse models serve as valuable tools for investigating sub-health, particularly its inflammatory and behavioral consequences [2,4], and for evaluating the efficacy of probiotic interventions in alleviating these effects [10].

*Bifidobacterium animalis* subsp. *lactis*, a recognized probiotic, exhibits anti-inflammatory and immunity-enhancing properties, resulting in its widespread use in health management and clinical settings [[11], [12], [13]]. *B. animalis* subsp. *lactis* supplementation has been shown to alleviate depression and anxiety in patients with coronary artery disease [14] and attenuate the impact of anticipatory stress on the immune system before night shift work [15], suggesting its potential benefits for individuals experiencing sub-health symptoms. However, its probiotic effects are strain-specific. For example, *B. animalis* subsp. *lactis* Bf141 significantly alleviates obesity and metabolic disorders while enhancing intestinal integrity; in contrast, the strain Bf26 failed to demonstrate similar effects [16]. Therefore, identifying specific *B. animalis* subsp. *lactis* strains with enhanced efficacy in alleviating health symptoms is of paramount importance.

Currently, limited data are available regarding the potential of *B. animalis* subsp. *lactis* in alleviating sub-health problems [10]. To address this knowledge gap, the present study aimed to screen and characterize probiotic strains of *B. animalis* subsp. *lactis* by collecting a diverse panel of isolates, performing whole-genome sequencing, and comprehensively characterizing their physiological and genomic traits at the strain level. Subsequently, we employed an SD-induced sub-health mouse model to evaluate the efficacy of the selected strains in alleviating inflammation and behavioral abnormalities. This study provides a valuable resource for characterizing *B. animalis* subsp. *lactis* strains and identifying promising candidates for alleviating sub-health symptoms, offering novel insights into the potential of this probiotic species for managing sub-health.

## 2 Materials and methods

### 2.1 Bacterial strain isolation and identification from fecal and probiotic samples

Fecal samples were obtained from a 1-month-old infant and a 50-year-old adult in China, while probiotic products were commercially sourced. The samples were processed within 6 h under strict anaerobic conditions (80 % N_2_, 10 % CO_2_, and 10 % H_2_). Following filtration (40-μm) and serial dilution, samples were plated on MRS agar (Qingdao Hope Bio-Technology Co., Ltd., Qingdao, China) supplemented with 0.05 % L-cysteine (w/v) and incubated anaerobically at 37 °C for 2–4 days using anaerobic culture bags containing AnaeroPack (Mitsubishi Gas Chemical, Tokyo, Japan). Single colonies were selected and inoculated into tubes containing MRS broth in an anaerobic glove box. After 2 days of cultivation, PCR amplification of the 16S rRNA gene was performed, followed by BLAST analysis (https://blast.ncbi.nlm.nih.gov/Blast.cgi) and phylogenetic analysis using MEGA 11.

### 2.2 Whole-genome sequencing and comparative genomic analysis

Bacterial cell pellets were collected and submitted to Magigene Tech Co., Ltd. (Shenzhen, China) for genomic DNA extraction and whole-genome sequencing using the Illumina NovaSeq PE150 (Illumina, Inc., San Diego, CA, USA) and Nanopore (Oxford Nanopore Technologies, Oxford, United Kingdom) platforms. High-quality genomes were assembled using Unicycler (v0.4.8). Genome completeness and contamination were assessed using CheckM (v1.1.3), which confirmed 100 % completeness and no contamination. The genomes of WLBA7 (HN019), WLBA11 (AD011), and WLBA13 (V9) were retrieved from the National Center for Biotechnology Information database, and the genomic data for the remaining strains were deposited in the National Microbial Data Center with accession number: NMDC10019713 (https://nmdc.cn/resource/genomics/project/detail/NMDC10019713).

Genome annotation was performed using Prokka (version 1.14.6) [17] with functional annotation against the Kyoto Encyclopedia of Genes and Genomes and protein family (Pfam) databases (pfam-legacy.xfam.org). Roary (version 3.13.0) was used to generate pan-genome and core-genome profiles [18]. Phylogenomic relationships were inferred using homology-averaged nucleotide identity (OrthoANI) analysis. A phylogenetic tree was constructed using FastTree and visualized using iTOL [19]. Enzymes involved in carbohydrate metabolism were annotated using carbohydrate-active enzymes (CAZymes) (dbCAN HMMdb version 12.0) [20]. Antibiotic resistance genes were predicted using the Comprehensive Antibiotic Resistance Database, with an identity threshold of 50 % [21].

### 2.3 Physiological and biochemical characterization

#### 2.3.1 Growth rate and carbon source utilization

Growth rates were determined by measuring the absorbance at 600 nm of 16 *B animalis* subsp. *lactis* cultures incubated at 37 °C in MRS broth within anaerobic tubes. The ability of *B. animalis* subsp*. lactis* to utilize different carbon sources was assessed using a 96-well BIOLOG ANI microplate (BIOLOG Inc., Hayward, CA, USA). Fresh cells grown overnight until the stationary phase were used to prepare bacterial suspensions in BIOLOG buffer. The suspensions were adjusted to appropriate concentrations and inoculated into microplates containing 95 carbohydrate substrates and a negative control. The difference in absorbance between 590 nm and 750 nm in each well was measured to determine the strain’s ability to utilize carbon sources after anaerobic incubation at 37 °C for 48 h.

#### 2.3.2 Antibiotic sensitivity

The antibiotic susceptibilities of *B. animalis* subsp. *lactis* strains were determined using the disk diffusion method [22]. Bacterial cells were spread onto MRS agar, and disks containing different antibiotics were placed firmly on the agar surface. The diameter of the inhibition zone was measured after 48 h of anaerobic incubation at 37 °C.

#### 2.3.3 Acidity/alkalinity tolerance

Sterile, anaerobic 1 × phosphate-buffered saline (PBS) solutions were prepared at pH values of 2.5, 3, 3.5, 7, and 9. Overnight cultures were harvested via centrifugation (6000 rpm, 5 min), and the supernatant was discarded. The bacterial cells were then resuspended in 1 mL of 1 × PBS with different pH values and incubated at 37 °C for 4 h. To neutralize the pH, the bacterial suspensions were serially diluted to 10^–6^ in anaerobic 1 × PBS at pH 7. Then, 50 μL of each dilution was spread onto MRS plates and cultured at 37 °C for 48 h in triplicate for each treatment per strain. The number of colony-forming units (CFU) on each plate was counted using an Automated Colony Counter (Shineso, China), and the survival rate was calculated using the plate at pH 7 as a control.

#### 2.3.4 Bile acid tolerance

Ten microliters of overnight cultures (approximately 10^7^–10^8^ CFU/mL) were spotted onto MRS agar plates supplemented with oxgall bile at concentrations of 0.1 %, 0.5 %, and 1 % (w/v, Yuanye, Shanghai, China) [23]. The plates were incubated anaerobically at 37 °C for 5 days. The minimum inhibitory concentration of oxgall bile for each strain was determined based on the absence of visible growth. Three replicates were analyzed for each strain at each bile concentration.

#### 2.3.5 Metabolite profiling

Volatile metabolite profiles were obtained using solid-phase microextraction (SPME) fibers (50/30 μm DVB/CAR/PDMS, Supelco, USA) and analyzed via gas chromatography-mass spectrometry (GC–MS) [24]. The metabolites were identified based on a minimum match score of 85 % against the National Institute of Standards and Technology library (NIST14; www.nist.gov) [25]. To isolate the metabolites specifically produced by the strains, the results were compared with those from blank MRS broth, and the compounds detected in the controls were excluded. The relative abundance of each metabolite is represented as the log10-transformed peak area.

The levels of short-chain fatty acids (SCFAs), including acetic, propionic, butyric, valeric, isobutyric, isovaleric, and hexanoic acid, were quantified using GC–MS (QP2010 Plus, JPN) because of its high sensitivity [24]. For sample preparation, 36-hour bacterial cultures and blank MRS broth were extracted with equal volumes of chromatographic-grade ethyl acetate (3 mL).

Lactic acid, succinic acid, and malic acid were quantified using an LC-MS system (Agilent 1260/6460, USA), with slight modifications to previously published methods [26,27] to minimize derivatization and enhance analyte recovery. For sample preparation, 500 μL of a 36-hour bacterial culture was centrifuged at 12,000 rpm for 5 min, and the supernatant was collected and filtered through a 0.22 μm-aqueous filter membrane.

### 2.4 Animals and experimental design

All animal procedures were performed in accordance with the Declaration of Helsinki and approved by the Ethics Committee of Shandong University (Approval No. SYDWLL-2023–063). Specific-pathogen-free female C57BL/6 J mice (5–6 weeks old) were obtained from Beijing Vital River Laboratory Animal Technology Co., Ltd. (Beijing, China) and housed under controlled environmental conditions (22–26 °C, 60–80 % humidity, 12-h light/dark cycle) with *ad libitum* access to sterile water and a standard chow diet.

To induce a sub-healthy model, the mice were subjected to sleep deprivation and intermittent light stimulation. Following a 7-day acclimation period, the mice were randomly assigned to five groups (*n* = 6/group). Before initiating the experimental intervention, the mice underwent a 7-day habituation period to the sleep deprivation apparatus with a daily exposure of 3 h. During weeks 2–11, the *B. animalis* subsp. *lactis*-treated and model groups were subjected to 23 h of daily sleep deprivation and light stimulation, followed by a 1-hour recovery period. Concurrently, the mice received daily oral gavage of *B. animalis* subsp. *lactis* (2 × 10^9^ CFU/day) or vehicle (PBS, control, and model groups).

### 2.5 Behavioral assessments

Behavioral testing was performed three days before the termination of the experimental period. Motor coordination and fatigue resistance were assessed using a rotarod test. Briefly, a rotarod was programmed to accelerate from 1 to 30 rpm for 60 s, and the latency to fall was recorded. Cognitive function and anxiety-like behaviors were evaluated using the Y-maze and elevated plus maze (EPM), respectively.

For the Y-maze test, the three arms were designated as “start” “novel” and “other” During the training phase, the novel arm was blocked, and mice were allowed to explore the maze from the start arm for 10 min. After a 2-h delay, the block was removed, and the mice were reintroduced to the start arm for a 5-min test period. The time spent and distance traveled in the novel arm were recorded using overhead video tracking.

For the EPM test, the mice were placed on the central platform and their behavior was recorded for 6 min following entry into a closed arm. The total number of arm entries, open arm entries, time spent in the open arms, and the percentage of time spent in the open arms were quantified using overhead video tracking and behavioral analysis software.

### 2.6 Serum cytokine analysis, qPCR, and gut microbiota profiling

Serum levels of biochemical parameters (IL-1β, TNF-α, IL-6, and MDA) were measured using enzyme-linked immunosorbent assay (ELISA) kits (Boshen Company, Nanjing, China) according to the manufacturer’s instructions.

Total RNA was extracted from colon tissue and hippocampal samples, and cDNA was synthesized using a HiScript III RT SuperMix (Vazyme Biotech, Nanjing, China). mRNA expression was normalized to that of glyceraldehyde-3-phosphate dehydrogenase (*GAPDH*) and calculated using the comparative threshold cycle (Ct) method. The qPCR primer sequences used are listed in Table S1a.

For gut microbiota profiling, total microbial DNA was extracted from fecal samples using a fecal bacterial DNA extraction kit (Tiangen, Beijing, China), and the V3-V4 regions of the 16S rRNA gene were amplified. Gut microbiota was sequenced by Benagen (Wuhan, China) and Personal Bio (Shanghai, China). The clean data were denoised (command: unoise3) and analyzed using Qiime2 (version: 2023.7) [28]. Linear discriminant analysis (LDA) effect size (LEfSe) was conducted to evaluate differences in gut microbiota composition between groups, which were analyzed using the OmicStudio online tool (version 2.1) [29].

### 2.7 Statistical analysis

Data from three independent experiments are presented as the mean ± standard error of the mean (SEM), and the overall statistical significance was determined using one-way analysis of variance (ANOVA), followed by Tukey’s post-hoc test for multiple comparisons to identify which treatment groups differed significantly. Statistical significance was indicated as follows: * *p* < 0.05, ** *p* < 0.01, and *** *p* < 0.001. All statistical analyses were performed using GraphPad Prism version 8 (GraphPad Software Inc., La Jolla, CA, USA).

## 3 Results

### 3.1 Comparative genomics and growth analysis of 16 B animalis subsp. lactis strains

**Fig. 1:**
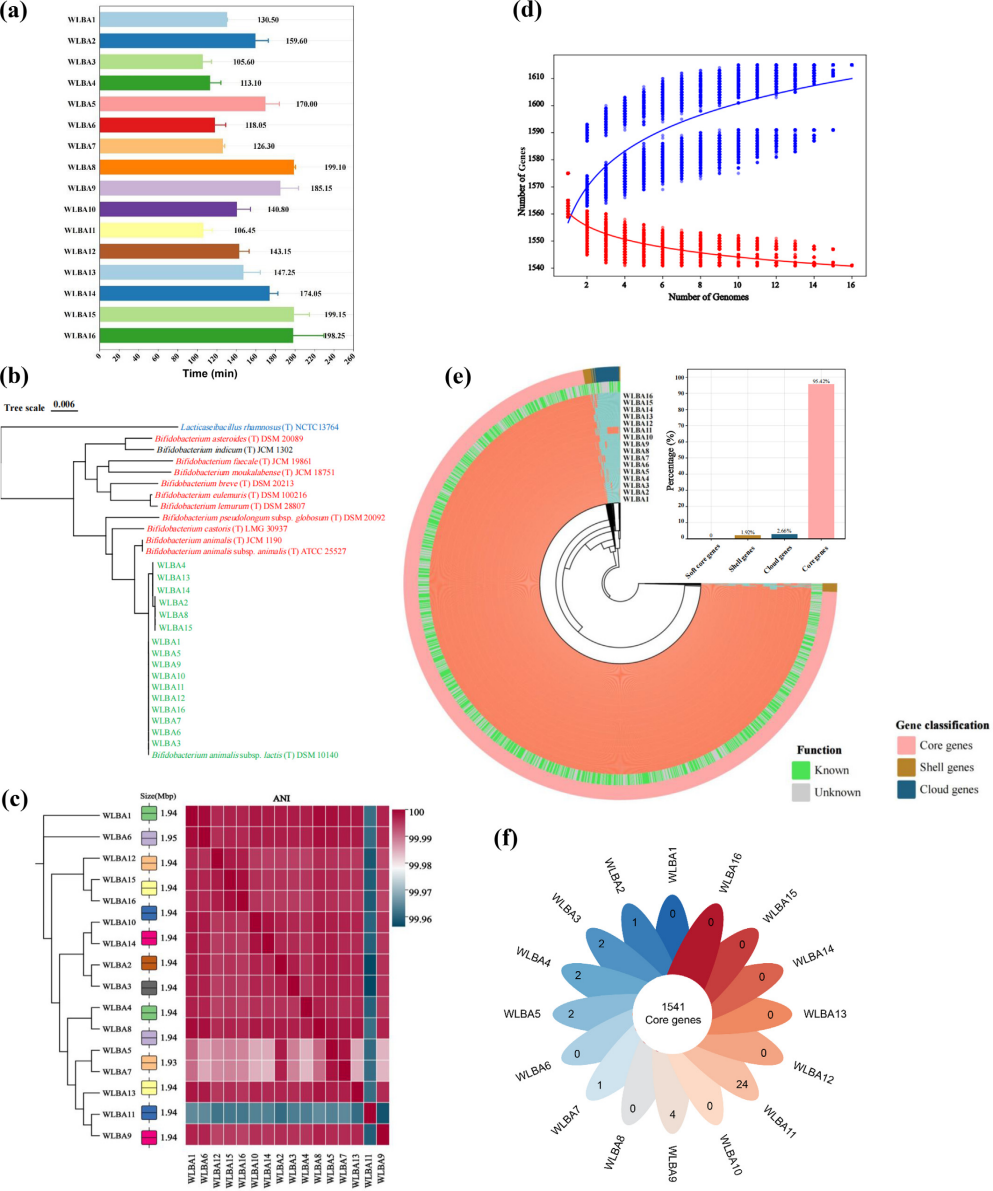
Growth and genomic characteristics of 16 *B animalis* subsp. *lactis* strains. (a) Generation time of 16 strains in MRS broth. (b) Phylogenetic relationship of the 16 strains and their close relatives based on 16S rRNA gene sequences. (c) Genome size and average nucleotide identity (ANI), and phylogenomic tree, constructed using core genome-based neighbor-joining method. (d) Pan-genome analysis illustrating the accumulation of new genes with each added genome (red: core-genome; blue: pan-genome). (e) Distribution of core genes, shell genes, and cloud genes in 16 genomes. Gene classification statistics (top right corner); pan-genome analysis: each ring represents a strain, and each radial extension within the ring corresponds to a specific gene (orange: present; blue: absent); the outer gray-green ring represents COG functional annotation, with gray indicating unknown functions and green indicating known functions; the outermost ring highlights specific gene sets: core genes in pink, shell genes in brown, and cloud genes in dark blue. (f) Venn diagram of the core and unique gene counts of 16 *B animalis* subsp. *lactis* strains*.*

In this study, we obtained two *B. animalis* subsp. *lactis* strains that were newly cultivated in human feces, along with 11 strains from probiotic samples and three strains from hGMB [30]. Growth rate analysis revealed that WLBA11 and WLBA3 had the shortest doubling times (106.45 and 105.60 min, respectively) (Fig. 1a). The superior growth performance of WLBA3 suggests its potential for rapid proliferation, which is a critical factor for probiotic strains. The genomes of the 16 strains were then sequenced and circularized, with sizes ranging from 1.93 to 1.95 Mb and GC content of 60.49 %. Phylogenetic analysis based on the 16S rRNA gene sequences revealed that the 16 strains were closely related (Fig. 1b). ANI analysis confirmed high genomic similarity among the 16 strains, with values ranging from 99.96 % to 99.99 % despite their diverse origins. Phylogenomic analysis indicated that WLBA1 and WLBA6 formed distinct phylogenetic branches that diverged from the predominant clusters (Fig. 1c).

We subsequently conducted a comparative genomic analysis of the 16 *B animalis* subsp. *lactis* strains. As shown in Fig. 1d, the cumulative curves of the pan- and core-genomes indicated that the pan-genome continued to grow, whereas the core-genome gradually decreased and tended to stabilize with increasing numbers of genomes. A pan-genome plot representing gene presence/absence across strains revealed gaps in regions lacking homologous sequences, consistent with the genome alignment map (Fig. 1e). Roary analysis revealed that the pan-genome of the 16 strains contained 1615 genes, of which 673 were annotated as hypothetical proteins. The petal plot indicates that the number of strain-specific genes ranged from 0 to 24. The core genes of *B. animalis* subsp. *lactis*, which were present in >95 % of the genomes, comprised 1541 genes; shell genes (present in 15–95 % of the genomes) and cloud genes (present in <15 % of the genomes) accounted for 29 (2 %) and 45 (3 %) genes, respectively (Fig. 1e-1f; Table S1b). The cloud genes included the β-galactosidase gene (*lacZ*) in WLBA4 and the 5,10-methyltetrahydrofolate reductase gene in both WLBA15 and WLBA16. Furthermore, Prokka annotation revealed that WLBA4 lacks a *GlnH* subunit, and Pfam annotation indicated that WLBA14 and WLBA10 lack the *Mur* glutamate ligase domain (Table S1c), which might affect their acid tolerance.

### 3.2 Physiological/biochemical characteristics and genetic basis of 16 B animalis subsp. lactis strains

**Fig. 2:**
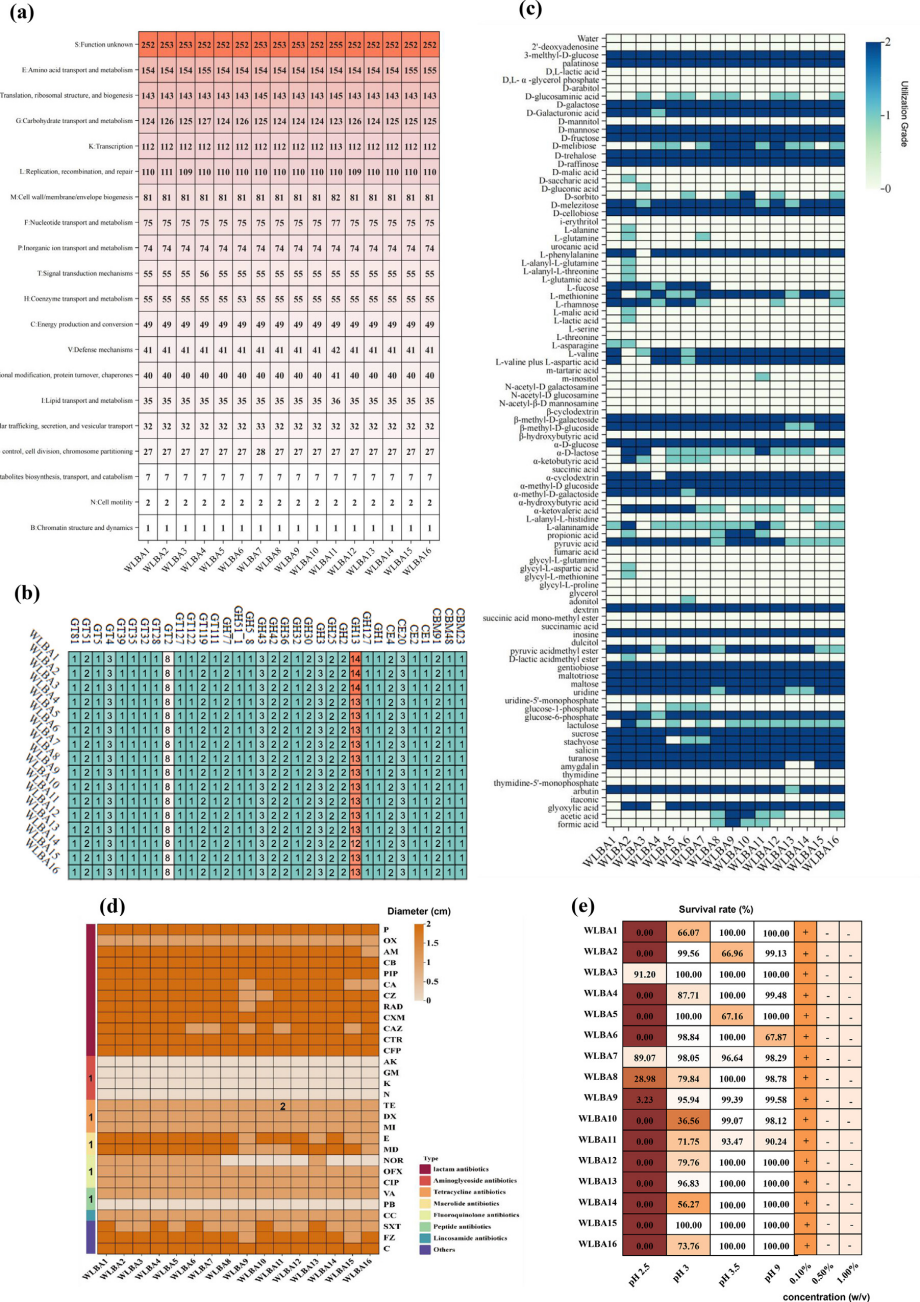
Genomic and physiological characterization of 16 *B animalis* subsp. *lactis* strains. (a) Genome annotation for Clusters of Orthologous Groups (COG) of 16 *B animalis* subsp. *lactis* genomes. (b) CAZyme categories in the 16 genomes. (c) Experimental determination of carbon source assimilation by 16 *B animalis* subsp. *lactis* strains. (d) Genome annotation and experimental determination of antibiotic resistances, the figures within the diagram depict the quantity of resistance genes. (e) Tolerances to acidity/alkalinity and bile acids. +: normal growth; −: growth was inhibited by the presence of ox gall bile.

To better understand the 16 *B animalis* subsp. *lactis* strains, we integrated genome annotation with analyses of carbon source assimilation, antibiotic resistance, and tolerance to acidic/alkaline conditions and bile acids. Genome annotation using COG analysis revealed a conserved set of core functional protein categories across all 16 genomes, with amino acid transport and metabolism (E), translation, ribosomal structure, and biogenesis (J), carbohydrate transport and metabolism (G), and transcription (K) being the most abundant. Although the numbers of proteins associated with amino acid transport and metabolism (E), nucleotide transport and metabolism (F), and inorganic ion transport and metabolism (P) were largely consistent, WLBA4, WLBA2, and WLBA6 exhibited a higher abundance of proteins related to carbohydrate transport and metabolism (G) (Fig. 2a). CAZyme analysis identified 34 carbohydrate-active enzyme modules across 16 genomes, including 14 glycoside hydrolases (GHs), 13 glycosyltransferases, 3 carbohydrate-binding modules, and 4 carbohydrate esterases (Fig. 2b). GH13 (α-amylase; EC 3.2.1.1), GT2 (cellulose synthase; EC 2.4.1.12), and GH3 (β-glucosidase; EC 3.2.1.21) exhibited the highest copy numbers. Carbohydrate metabolism-related genes, including those of the GH13 family, were generally conserved; however, WLBA1, WLBA2, and WLBA3 possessed 1–2 additional copies of GH13 compared to the other strains (Fig. 2b).

BIOLOG assays revealed that all strains effectively utilized a range of carbon sources (28), including α-D-glucose, D-galactose, D-galacturonic acid, maltotriose, D-mannose, and turanose. Consistent with the presence of GH1 family genes, all strains effectively utilized D-mannose, while the strains’ utilization of maltose and gentiobiose closely matched the presence of GH13 family genes (Fig. 2c). However, strain-specific differences in carbon source utilization were evident, which did not fully correlate with the gene annotation results. For example, WLBA2, WLBA6, WLBA7, and WLBA3 utilized 53, 46, 46, and 44 carbon sources, respectively, whereas WLBA4 and WLBA15 utilized only 39 and 38 carbon sources, respectively (Fig. 2c). Despite possessing abundant GH13 family genes, strains such as WLBA8 and WLBA9 could not assimilate L-fucose, suggesting strain-level diversity in carbon source utilization among the 16 *B animalis* subsp. *lactis* strains investigated in this study.

As potential probiotics, we investigated the tolerance of the 16 *B animalis* subsp. *lactis* strains to acidic/alkaline environments and in the presence of bile acids, as well as their antibiotic resistance. Genome analysis identified 10 distinct antibiotic resistance genes across the 16 genomes, conferring resistance to elfamycin, fluoroquinolones, macrolides, peptides, rifamycin, streptogramins, tetracyclines, aminocoumarins, fusidanes, and mupirocin-like compounds. Although the types of resistance genes were consistent among the strains, the number of coding genes varied. For instance, WLBA11 carried a higher number of tetracycline resistance genes than other strains (Fig. 2d). According to the Kirby–Bauer method, all 16 strains exhibited consistent antibiotic resistance profiles (Fig. 2d). The strains were tolerant to five antibiotics (amikacin, gentamicin, kanamycin, neomycin, and polymyxin B), but sensitive to β-lactams, including penicillin. The observed resistance to polymyxin aligned with the detection of peptide antibiotic resistance genes in all strains. Although all strains were predicted to harbor tetracycline resistance genes, phenotypic tests revealed sensitivity to tetracycline (Fig. 2d), highlighting the potential discrepancy between genomic predictions and phenotypic resistance. Furthermore, all 16 strains demonstrated robust tolerance to both acidic (pH 3.5; 14 strains >93 % survival) and alkaline (pH 9.0; 15 strains >90 % survival) conditions, exhibiting functional concordance with the pan-genomic analysis. Regarding bile acid tolerance, all strains grew normally at 0.1 % oxgall bile conditions but were significantly inhibited in 0.5 % ox gall bile conditions (Fig. 2e).

### 3.3 Strain-level metabolic profiling of 16 B animalis subsp. lactis strains

**Fig. 3:**
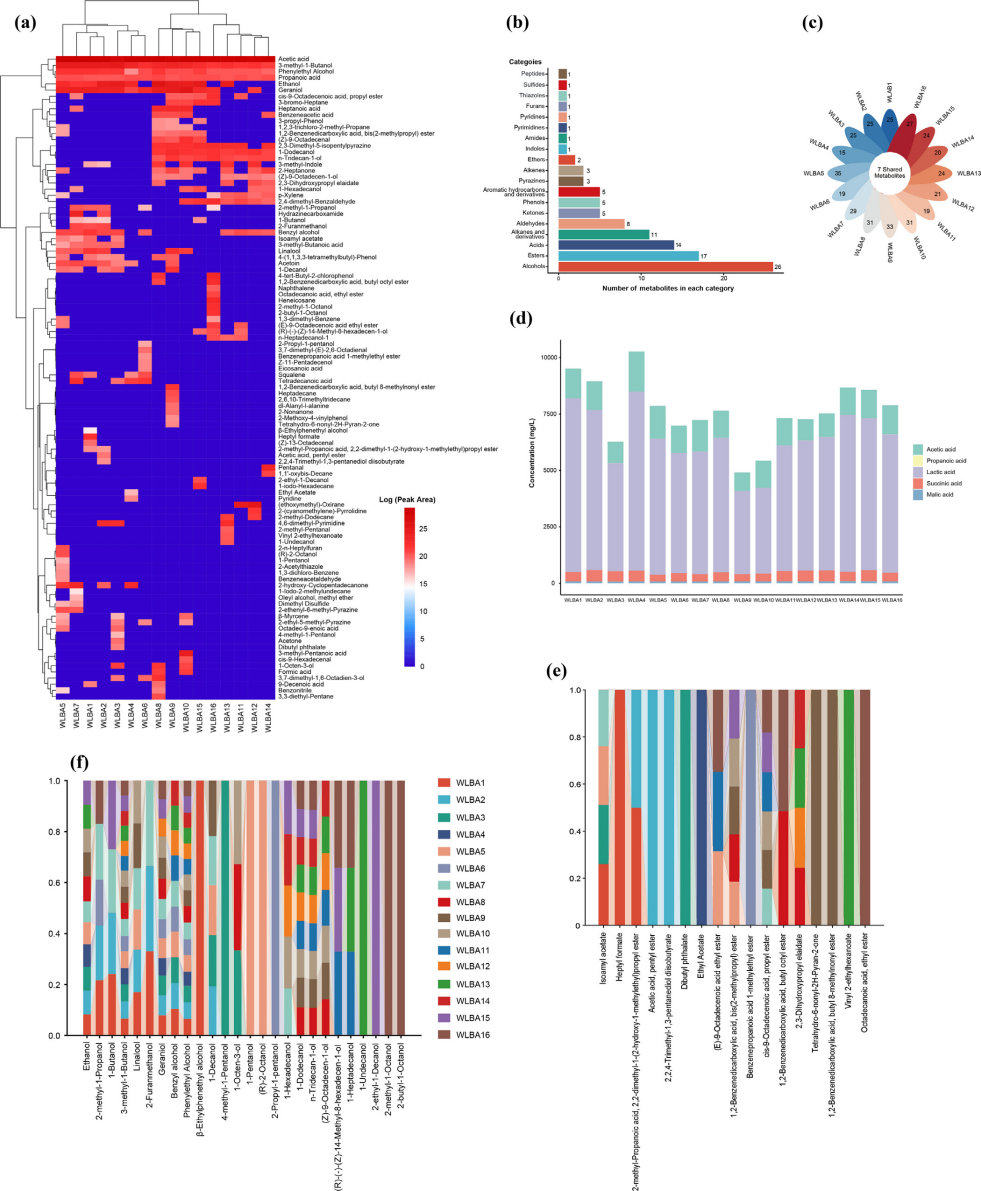
Metabolic profiling of 16 strains of *B. animalis* subsp. *lactis*. (a) Summary of 104 non-redundant volatile metabolites. (b) Categories of all metabolites. (c) Core (shared) and unique metabolites. (d) Production of SCFAs and lactic, succinic, and malic acids. (e) Count of specified ester compounds. (f) Count of specified alcohol compounds.

We employed SPME/GC–MS to profile the volatile metabolites produced by the 16 *B animalis* subsp. *lactis* strains investigated. A total of 104 unique compounds were identified (lactic, succinic, and malic acids were confirmed via LC-MS), with individual strains producing 15–35 metabolites (Fig. 3a). These metabolites were classified into 18 groups: alcohols (*n* = 26), esters (*n* = 17), acids (*n* = 11), alkanes and their derivatives (*n* = 11), and aldehydes (*n* = 8), among others (Fig. 3b). WLBA5 produced the most metabolites (35), followed by WLBA9 (33). Notably, the 16 strains shared a core set of seven compounds, comprising two alcohols (3-methyl-1-butanol and phenethyl alcohol) and five acids (lactic, succinic, malic, acetic and propionic acids) (Fig. 3c). The production of these five acids was further assessed, revealing that the strains primarily produced acetic and lactic acid (Fig. 3d). Quantitative GC–MS analysis showed that WLBA4 and WLBA5 produced higher levels of acetic acid (1775 and 1453 mg/L, respectively) than WLBA9, while WLBA12 exhibited lower production (822 and 944 mg/L, respectively). Interestingly, 3-methylbutanoic acid (isovaleric acid) was detected in five *B. animalis* subsp. *lactis* strains, but was not quantified via GC–MS, likely due to concentrations below the detection limit. Although present in low abundance, this metabolite may exert beneficial effects on the gut microenvironment at physiological concentrations.

LC-MS analysis revealed that all 16 *B animalis* subsp. *lactis* strains produced substantial amounts of lactic acid, with notable inter-strain variation. WLBA4 and WLBA1 exhibited the highest lactic acid production of 7936 and 7695 mg/L, respectively. Theoretically, the phosphoketolase pathway, which leads to heterolactic fermentation in *Bifidobacterium* species, generates lactic and acetic acids at a stoichiometric molar ratio of 2:3. This characteristic is consistent with the high production of lactic and acetic acids observed in the 16 *B animalis* subsp. *lactis* strains, although the ratio did not conform to the theoretical 2:3 ratio. In addition to lactic and acetic acids, succinic and malic acids were also produced by the 16 strains. WLBA2 and WLBA15 exhibited the highest levels of succinic acid production (548 and 532 mg/L, respectively), whereas WLBA5 and WLBA9 produced the lowest levels (342 and 359 mg/L, respectively). Malic acid production varied across the 16 strains, ranging from 85 mg/L in WLBA3 to 103 mg/L in WLBA14 (Fig. 3d). In addition to lactic and acetic acids, the production of succinic and malic acids suggests the involvement of alternative metabolic routes contributing to their synthesis.

We further investigated esters and alcohols among the categories of metabolites detected. We detected 17 distinct esters (Fig. 3e), 9 of which showed strain-specific production: heptyl formate (WLBA1), pentyl acetate (WLBA2), dibutyl phthalate (WLBA3), ethyl acetate (WLBA4), 1-methyl ethyl phenylpropanoate (WLBA6), butyldecanolide and 8-methylnonyl butyl phthalate (WLBA10), 2-ethylhexyl acrylate (WLBA13), and ethyl stearate (WLBA16). Meanwhile, 26 different alcohols (C2-C17) were detected (Fig. 3f), with 9 also showing strain-specific production: β-ethylphenethyl alcohol (WLBA1), 4-methyl-1-pentanol (WLBA3), 1-pentanol and (*R*)-2-octanol (WLBA5), 2-propyl-1-pentanol (WLBA6), 1-undecanol (WLBA13), 2-ethyl-1-decanol (WLBA15), and 2-methyl-1-octanol and 2‑butyl‑1-octanol (WLBA16).

To further explore the potential of *B. animalis* subsp. *lactis* to affect sub-health conditions, WLBA3, WLBA7, and WLBA6 were selected based on their distinct phenotypic and genotypic profiles. Specifically, WLBA3 was prioritized for its potential to survive and proliferate in the gastrointestinal tract, as evidenced by its robust tolerance to acidic/alkaline conditions, rapid growth rate, and ability to produce beneficial metabolites, such as isovaleric acid. WLBA7 was selected because of its strong acid-alkali tolerance, market prevalence, and ability to produce isovaleric acid and other metabolites. WLBA6, representing a distinct phylogenetic clade and exhibiting a higher abundance of genes encoding proteins involved in carbohydrate transport and metabolism, as determined by genome annotation, was included to enhance phylogenetic diversity in this study.

### 3.4 B animalis subsp. lactis alleviates inflammation, anxiety-like behavior, cognitive function, and exercise fatigue in sub-health mice

**Fig. 4:**
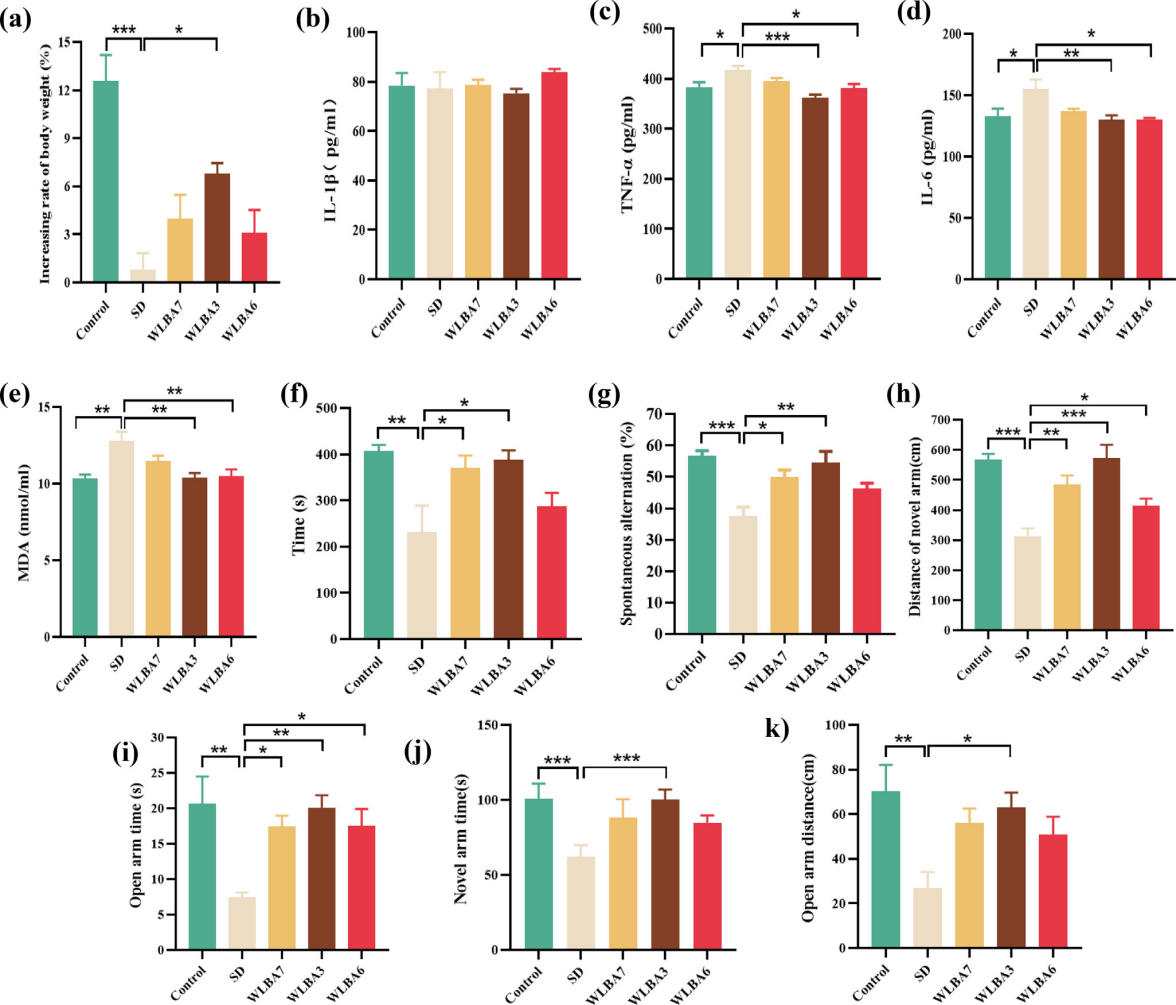
*B. animalis* subsp. *lactis* strains alleviates sub-health symptoms including inflammatory factors, cognitive function, and anxiety like behavior in mice (*n* = 6/group). (a) Percentage of weight gain during SD on the last day. ELISA analyses of (b) IL-1β; (c) TNF-α; (d) IL-6; (e) MDA in serum. (f) Endurance time on the rotarod. Y-maze: (g) spontaneous alternation rate; (h) distance of motion in novel arm; (i) the time spent in novel arms. EPM: (j) distance of motion in open arms; (k) the time spent in open arms.

Compared to non-SD controls, SD mice exhibited a significant reduction in body mass and elevated serum levels of TNF-α, IL-6, and MDA, while IL-1β levels remained unchanged (Fig. 4a-4e). The oral administration of WLBA3 or WLBA6 attenuated the SD-induced body mass loss (Fig. 4a) and elevations in TNF-α (Fig. 4c), IL-6 (Fig. 4d), and MDA levels (Fig. 4e). In contrast, WLBA7 exhibited no significant effect (Fig. 4a-4e). Notably, WLBA3 demonstrated the most potent anti-inflammatory and antioxidant effects, effectively suppressing the elevated levels of TNF-α, IL-6, and MDA to levels comparable to, or even lower than, those of the control group (Fig. 4c-4e).

The effect of SD on physical performance was assessed using the rotarod test. SD mice exhibited a significant decrease in latency to fall compared to controls, indicating reduced endurance. Supplementation with WLBA3 and WLBA7 significantly increased the time spent by the SD mice on the rotarod (Fig. 4f), demonstrating the anti-fatigue effects of these strains.

Cognitive function in the SD mice was evaluated using the Y-maze test. Compared to controls, SD mice exhibited a significantly reduced spontaneous alternation rate, indicating impaired spatial working memory. Treatment with *B. animalis* subsp. *lactis* restored spontaneous alternation, with WLBA3 demonstrating the most robust effect (Fig. 4*g*). Further assessment of learning and memory via the Y-maze novel arm recognition test revealed a significant reduction in both the distance traveled and number of entries into the novel arm in the SD group, suggesting impaired learning and memory. Administration of WLBA3 reversed these deficits, increasing both the distance traveled and the time spent in the novel arm (Fig. 4h-4i), thereby demonstrating its capacity to ameliorate cognitive dysfunction.

Finally, the EPM test revealed that anxiety-like behavior observed in SD mice was significantly alleviated by both WLBA3 and WLBA6. Following treatment with these strains, both the movement distance and duration in the open arms significantly increased (Fig. 4j-4k), further supporting the anxiolytic properties of these strains.

### 3.5 WLBA3-mediated modulation of the NLRP3 inflammasome and gut microbiota

**Fig. 5:**
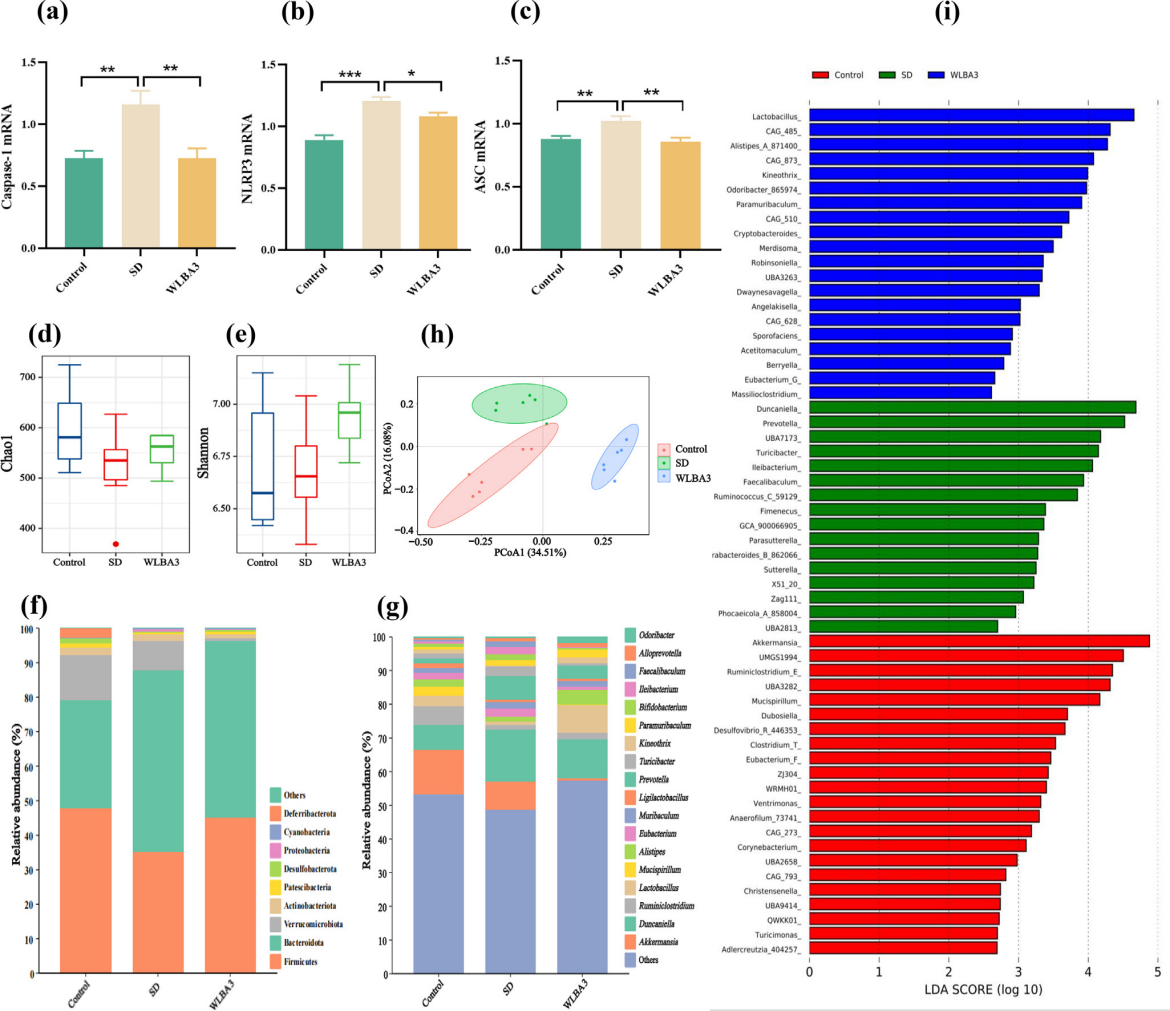
Effects of WLBA3 on NLRP3 inflammasome activation and gut microbiota. (a) RT-PCR of hippocampal Caspase-1 mRNA normalized to expression of GAPDH, (b) NLRP3, (c)ASC, normalized to expression of GAPDH. (d) Chao1 index, and (e) Shannon index of gut microbiota; (f) Microbial taxa at the phylum level. (g) The top 15 microbial taxonomic groups in terms of genus level. (h) PCoA result. (i) LEfSe results (LDA>2).

Given the observed anti-inflammatory and antioxidant effects of WLBA3, we investigated the mechanisms by which this strain improves the cognitive and behavioral performance of SD mice. RT-qPCR analysis of hippocampal tissue revealed that WLBA3 treatment significantly attenuated the mRNA expression of the key NLRP3 inflammasome components *NLRP3, ASC*, and caspase-1 compared to the SD group (Fig. 5a-5c). These data suggest that WLBA3 alleviates neuroinflammation by inhibiting the NLRP3 inflammasome pathway, thereby ameliorating the cognitive and behavioral deficits in SD-induced sub-healthy mice.

Analysis of gut microbiota α-diversity revealed a trend toward restoration of the Chao index in the WLBA3 group, although neither Chao nor Shannon indices reached statistical significance (Fig. 5d-5e). At the phylum level, SD induced a decrease in the abundance of Firmicutes and a corresponding increase in that of *Bacteroidetes*; these effects were reversed by WLBA3 treatment (Fig. 5f). At the genus level, SD resulted in the depletion of *Lactobacillus* and *Blautia* and the enrichment of *Prevotella* and *Duncaniella*; WLBA3 treatment significantly reversed these alterations (Fig. 5g). Principal coordinate analysis demonstrated the clear segregation of gut microbial communities in the SD, control, and WLBA3 groups (Fig. 5h). LEfSe analysis identified the enrichment of *Lactobacillus* and *Alistipes* following WLBA3 treatment, whereas *Ileibacterium* was enriched in the SD group (Fig. 5i). These findings indicate that both SD and WLBA3 exert significant modulatory effects on the gut microbial community structure in mice, with WLBA3 primarily acting to restore beneficial bacteria altered by SD.

## 4 Discussion

Sub-health, an intermediate state between wellness and disease, poses a significant global health burden due to its high prevalence [1,2]. Consequently, the development and implementation of innovative strategies are crucial for mitigating this growing concern, including exploring the potential of probiotics to modulate the gut-brain axis and alleviate sub-health symptoms. Probiotic strain resources represent a core competitive advantage in the food, pharmaceutical, and agricultural sectors. As the catalog of the identified *B. animalis* subsp. *lactis* strains continues to expand, accumulating evidence underscores the importance of strain specificity, as demonstrated by divergent outcomes in disease resistance [31] and clinical trials for gastrointestinal disorders [[32], [33], [34], [35]]. Recognizing the critical role of strain selection, this study comprehensively investigated the *in vitro* physiological and biochemical properties of 16 *B animalis* subsp*. lactis* strains and provided a preliminary exploration of their strain-specific characteristics.

To exert their beneficial effects, probiotics must reach the colon in a viable state and efficiently colonize the ecological niche through rapid proliferation [36,37]. Rapid cell growth is a basic requirement of probiotic strains. Our results showed that WLBA3 exhibited a remarkably short generation time of only 2 h, suggesting robust *in vivo* colonization potential. However, survival in the gastrointestinal tract presents a significant challenge owing to its extreme pH conditions. Prokka annotation revealed that WLBA4 lacks the *GlnH* subunit, a known determinant of acid tolerance [38]. Similarly, Pfam annotation revealed that WLBA14 and WLBA10 lack the glutamate ligase domain of Mur, a gene essential for peptidoglycan synthesis and cell wall formation [39], which may compromise acid tolerance and growth. Conversely, WLBA3 and WLBA7, possessing these genes, demonstrated >89 % survival at pH 2.5 and 9.0. Furthermore, despite the high genomic similarity (ANI > 99.96 %) among the 16 strains, our findings demonstrated that experimental results can diverge significantly from genomic predictions, particularly in cases involving antibiotic resistance. This emphasizes the need for caution when interpreting genomic data and highlights the critical role of strain-level variations in determining functional traits.

Specific metabolites may be key molecules that mediate the beneficial effects of certain strains. For instance, WLBA3, WLBA5, and WLBA7 produce isovaleric acid, a branched-chain SCFA known to inhibit osteoporosis [40]. This metabolite may be absorbed by colonocytes via transporters such as SLC26A3/MCT1 and subsequently utilized as an energy source [41]. Furthermore, we observed that WLBA8 and WLBA9 produced phenylacetic acid, a crucial regulatory metabolite within the phenylalanine metabolic pathway, which attenuated epithelial-mesenchymal transition activity [42], warranting further investigation. Notably, 13 *B animalis* subsp. *lactis* strains produced geraniol, an acyclic monoterpene derivative. Despite the relatively low bioavailability of geraniol, reports have indicated that it has antibacterial, anti-inflammatory, and neuroprotective properties [43]. Our ongoing research aims to elucidate the underlying mechanisms of these effects. Collectively, our experiments, which were designed for rational strain selection, highlighted the probiotic potential of *B. animalis* subsp. *lactis* strains, particularly WLBA3 and WLBA7.

Limited evidence suggests that *B. animalis subsp. lactis* administration may alleviate sub-health symptoms, such as depression, anxiety, and immune dysregulation [14,15]. Growing interest has emerged in *B. animalis* subsp. *lactis* can enhance learning and memory functions [44]. In our SD-induced mouse model, WLBA3 not only reduced the levels of pro-inflammatory cytokines IL-6 and TNF-α but also significantly decreased the relative mRNA expression levels of *NLRP3, ASC*, and caspase-1, indicating its potential to alleviate neuroinflammation via the NLRP3 inflammasome pathway [45]. Behavioral analyses revealed that WLBA3 treatment effectively enhanced spatial working memory and reduced anxiety-like behaviors in mice exhibiting suboptimal health, as evidenced by a significant increase in the spontaneous alternation rate in the Y-maze test, dwell time, and entry into the open arms in the EPM test. Furthermore, WLBA3 treatment preserved the α-diversity of the gut microbiota while concurrently inducing significant compositional shifts, with a notable increase in the abundance of *Lactobacillus*. This genus is associated with improved sub-health outcomes and contains species such as *Limosilactobacillus reuteri*, which are known to alleviate anxiety-like behaviors [46]. Consistent with our results, studies have reported that the abundance of *Alistipes* increases following the administration of antidepressants, such as fluoxetine and amitriptyline [47,48]. These findings, combined with our results, suggest that WLBA3 modulates the gut microbiota by influencing carbohydrate metabolism and antibiotic resistance within the gut microbiota.

This study was limited by the relatively small number of *B. animalis* subsp. *lactis* strains collected and characterized, which may not fully represent the natural diversity of this subspecies. Future research should expand the strain collection to better capture this diversity. Although the current findings provide compelling evidence for the beneficial effects of WLBA3 on sub-health symptoms, further investigation is warranted to elucidate the underlying molecular mechanisms. Specifically, studies should examine the role of metabolites, such as isovaleric acid and geraniol, in mediating the observed alleviation effects. The small sample size (*n* = 6/group) in the animal experiments also warrants caution regarding our preliminary findings, which require confirmation in larger cohorts. Longitudinal studies are essential to assess the long-term impact of WLBA3, and well-designed clinical trials are required to validate the efficacy and safety of WLBA3 for translational applications in human health.

## 5 Conclusion

This study provides a comprehensive phenotypic and genomic characterization of 16 *B animalis* subsp. *lactis* strains, revealing conserved genetic backgrounds and significant inter-strain variability in key physiological traits. Notably, using an SD-induced sub-healthy mouse model, we demonstrated that the strain WLBA3 effectively alleviated weight loss, fatigue, and anxiety-like behavior while enhancing exercise endurance and cognitive function. These beneficial effects were associated with the modulation of the gut microbiota and inhibition of the NLRP3 inflammasome pathway. These findings underscore the significant potential of WLBA3 as a probiotic to alleviate fatigue, anxiety, and other sub-health symptoms.

## Data Availability Statement

The genomic data in this study were is available at the National Microbiology Data Center (https://nmdc.cn/resource/genomics), with Project Accession Number NMDC10019713.

## CRediT authorship contribution statement

**Yao-Kun Zhang:** Writing – original draft, Visualization, Validation, Resources, Investigation, Formal analysis, Data curation. **Liang Zhang:** Writing – original draft, Visualization, Investigation, Funding acquisition, Formal analysis, Data curation. **Xue Ni:** Investigation, Data curation. **Shu-Wen Zhang:** Investigation, Data curation. **Min-Zhi Jiang:** Methodology, Investigation, Data curation. **Si-Lu Zhang:** Supervision, Conceptualization. **Guo-Xun Xiao:** Supervision, Conceptualization. **He Jiang:** Resources. **Ming-Xia Bi:** Supervision, Methodology. **Yu-Lin Wang:** Supervision, Formal analysis. **Chang Liu:** Supervision, Conceptualization. **Shuang-Jiang Liu:** Writing – review & editing, Supervision, Project administration, Methodology, Funding acquisition, Conceptualization.

## Declaration of Competing Interest

The authors declare the following financial interests/personal relationships which may be considered as potential competing interests: Given their roles as Executive Guest Editor and Guest Editor, respectively, Dr. Shuangjiang Liu and Dr. Chang Liu had no involvement in the peer review of this article, and had no access to information regarding its peer review. Full responsibility for the editorial process for this article was delegated to Dr. Mudasir Dar.

Yan-Yi Zheng, Si-Lu Zhang, and Guo-Xun Xiao are staff members of the WONDERLAB Innovation Centre for Healthcare, an organization which is dedicated to the innovation and commercialization of probiotics and nutrition supplements and which has also partly supported this research. The funders had no role in the decision to publish this study.

## Appendix Supplementary materials

Image, application 1
