# Supplementary material for: Bifidobacterium animalis subsp. lactis genome resources and metabolite profiling at the strain level and their ability to alleviate anxiety-like behavior in a sleep-deprived mouse model
Source: Eng Microbiol. 2025 Jul 30;5(4):100228. doi: 10.1016/j.engmic.2025.100228 (PMC12967841; doi:10.1016/j.engmic.2025.100228)
Supplement: Supplementary file 1 [file mmc1.pdf]

The sequence of primers used for RT-qPCR

Gene	Forward Primer (5'-3')	Reverse Primer (5'-3')
GAPDH	CATCACTGCCACCCAGAAGACTG	ATGCCAGTGAGCTTCCCGTTCAG
Nlrp3	TCACAACTCGCCCAAGGAGGAA	AAGAGACCACGGCAGAAGCTAG
caspasa-1	GGCACATTTCCAGGACTGACTG	GCAAGACGTGTACGAGTGGTTG
ASC	CTGCTCAGAGTACAGCCAGAAC	CTGTCCTTCAGTCAGCACACTG

Gene	Non-unique Gene name	Annotation	Upstream gene	Downstream gene	SK-1	SK-2	SK-3	SK-4	SK-5	SK-6	SK-7	SK-8	SK-9	SK-10	SK-11	SK-12	SK-13	SK-14	SK-15	SK-16
group_116		hypothetical protein	Transcription-repair-coupling factor	Ribosomal RNA small subunit methyltransferase D	+	+	+	+	+	+	+	+	+	+	-	+	+	+	+	+
rpmG2		50S ribosomal protein L33 2			+	+	+	+	+	+	+	+	+	+	-	+	+	+	+	+
dasA		Diacetylchitobiose binding protein DasA			+	+	+	+	+	+	+	+	+	+	-	+	+	+	+	+
group_119		hypothetical protein	Glucosyl-3-phosphoglycerate phosphatase	tRNA-Ala(ggc)	+	+	+	+	+	+	+	+	+	+	-	+	+	+	+	+
group_121		hypothetical protein	Putative HMP/thiamine import ATP-binding protein YkoD	O-acetyl-L-homoserine sulfhydrylase	+	+	+	+	+	+	+	+	+	+	-	+	+	+	+	+
group_122		hypothetical protein	Formate acetyltransferase 1	Glutamine-dependent NAD(+) synthetase	+	+	+	+	+	+	+	+	+	+	-	+	+	+	+	+
group_123		hypothetical protein	Pyrimidine-specific ribonucleoside hydrolase RihA	Decaprenyl diphosphate synthase	+	+	+	+	+	+	+	+	-	+	+	+	+	+	+	+
group_120		hypothetical protein	Aliphatic sulfonates import ATP-binding protein SsuB	Transcription-repair-coupling factor	+	+	-	+	+	+	+	+	+	+	-	+	+	+	+	+
nadE_2		Glutamine-dependent NAD(+) synthetase			+	+	+	+	-	+	-	+	+	+	+	+	+	+	+	+
nadE_3		Glutamine-dependent NAD(+) synthetase			+	+	+	+	-	+	-	+	+	+	+	+	+	+	+	+
group_129		hypothetical protein	Glutamine-dependent NAD(+) synthetase	5S ribosomal RNA	+	+	+	+	-	+	-	+	+	+	+	+	+	+	+	+
treC		Trehalose-6-phosphate hydrolase			+	-	-	+	+	+	+	+	+	+	-	+	+	+	+	+
group_115		hypothetical protein	Aromatic amino acid transport protein AroP	putative ABC transporter ATP-binding protein	+	+	+	+	+	-	+	-	+	+	+	+	-	+	+	-
group_124		hypothetical protein	hypothetical protein	hypothetical protein	+	+	+	+	+	-	-	+	+	+	-	+	-	+	+	+
group_1545		ISL3 family transposase IS2001			+	+	+	+	+	-	-	+	+	-	+	+	+	+	-	-
group_1599		hypothetical protein	Oligo-1,6-glucosidase	ISL3 family transposase IS2001	-	+	-	+	+	-	+	+	-	+	+	+	-	+	-	+
group_113		hypothetical protein	hypothetical protein	Isoleucine-tRNA ligase	+	+	-	+	-	+	-	+	+	-	-	-	+	+	-	+
group_1543		ISL3 family transposase IS2001			-	+	+	+	-	+	-	+	+	-	-	+	+	+	-	-
group_125		hypothetical protein	hypothetical protein	hypothetical protein	+	+	-	-	-	+	-	+	+	-	-	+	-	-	+	+
group_1	malL_1	hypothetical protein	ISL3 family transposase IS2001	hypothetical protein	-	-	-	+	-	-	+	+	-	-	-	+	-	+	+	+
group_1598		hypothetical protein	Oligo-1,6-glucosidase	ISL3 family transposase IS2001	+	+	+	-	-	+	-	-	+	-	-	-	+	-	+	-
group_11		ISL3 family transposase IS2001			+	-	-	-	+	-	+	-	-	+	-	-	-	-	-	+
treC_1		Trehalose-6-phosphate hydrolase			-	+	+	-	-	+	-	-	+	-	-	-	+	-	-	-
group_3	malL_1	hypothetical protein	hypothetical protein	hypothetical protein	-	-	-	+	-	-	+	-	-	-	-	+	-	-	+	+
group_4	malL_1	Oligo-1,6-glucosidase			-	-	-	+	-	-	-	+	-	-	-	+	-	+	-	+
group_114		hypothetical protein	hypothetical protein	hypothetical protein	-	-	-	-	-	+	+	-	-	-	+	-	+	-	-	-
group_126		hypothetical protein	DNA-binding protein HB1	hypothetical protein	+	-	-	-	-	-	-	-	-	-	-	+	-	+	-	-
group_7	malL_1	Oligo-1,6-glucosidase			+	-	-	-	+	-	-	-	-	+	-	-	-	-	-	-
group_8		ISL3 family transposase IS2001			-	-	-	-	-	-	+	-	-	+	-	-	-	-	+	-
group_10		ISL3 family transposase IS2001			-	-	-	-	-	+	+	-	-	-	-	-	-	-	-	-
group_13		ISL3 family transposase IS2001			-	-	-	-	-	-	+	-	-	-	-	-	-	-	+	-
group_14		hypothetical protein	ISL3 family transposase IS2001	Trehalose-6-phosphate hydrolase	-	-	-	-	+	-	-	-	-	+	-	-	-	-	-	-
treC_2		Trehalose-6-phosphate hydrolase			-	+	-	-	-	-	-	-	-	-	+	-	-	-	-	-
group_16		hypothetical protein	hypothetical protein	hypothetical protein	-	+	+	-	-	-	-	-	-	-	-	-	-	-	-	-
metF_2		5,10-methylenetetrahydrofolate reductase			-	-	-	-	-	-	-	-	-	-	-	-	-	-	+	+
group_5	malL_2	Oligo-1,6-glucosidase			-	+	+	-	-	-	-	-	-	-	-	-	-	-	-	-
group_6	glgE	Trehalose-6-phosphate hydrolase			-	-	+	-	-	-	-	-	-	-	+	-	-	-	-	-
group_9		ISL3 family transposase IS2001			-	-	-	-	-	-	-	-	-	-	-	-	-	-	+	+
group_12		ISL3 family transposase IS2001			-	-	-	-	+	-	-	-	-	-	-	-	-	-	-	-
group_130		hypothetical protein	Tryptophan-tRNA ligase 2	hypothetical protein	-	-	-	-	-	-	-	-	-	-	+	-	-	-	-	-
group_131		hypothetical protein	Ketol-acid reductoisomerase (NADP(+))	hypothetical protein	-	-	-	-	-	-	-	-	-	-	+	-	-	-	-	-
group_132		hypothetical protein	Transcription-repair-coupling factor	Ribosomal RNA small subunit methyltransferase D	-	-	-	-	-	-	-	-	-	-	+	-	-	-	-	-
group_133		hypothetical protein	hypothetical protein	Lactose-binding protein	-	-	-	-	-	-	-	-	-	-	+	-	-	-	-	-
group_134		hypothetical protein	ATP-dependent RNA helicase DbpA	putative glucose uptake protein GlcU	-	-	-	-	-	-	-	-	-	-	+	-	-	-	-	-
group_135		hypothetical protein	Bifunctional (p)ppGpp synthase/hydrolase RelA	Peptidyl-prolyl cis-trans isomerase B	-	-	-	-	-	-	-	-	-	-	+	-	-	-	-	-
group_136		hypothetical protein	Glutamine-dependent NAD(+) synthetase	Formate acetyltransferase 1	-	-	-	-	-	-	-	-	-	-	+	-	-	-	-	-
group_137		hypothetical protein	Single-stranded DNA-binding protein	Neutral endopeptidase	-	-	-	-	-	-	-	-	-	-	+	-	-	-	-	-
group_138		hypothetical protein	hypothetical protein	Universal stress protein	-	-	-	-	-	-	-	-	-	-	+	-	-	-	-	-
group_139		hypothetical protein	ATP-dependent Clp protease ATP-binding subunit ClpC1	Aspartate-tRNA(Asp/Asn) ligase	-	-	-	-	-	-	-	-	-	-	+	-	-	-	-	-
group_140		hypothetical protein	16S ribosomal RNA	tRNA-Val(gac)	-	-	+	-	-	-	-	-	-	-	-	-	-	-	-	-
group_141		hypothetical protein	hypothetical protein	hypothetical protein	-	-	+	-	-	-	-	-	-	-	-	-	-	-	-	-
group_142		hypothetical protein	hypothetical protein	Vitamin B12 import ATP-binding protein BtuD	-	-	-	-	-	-	-	-	+	-	-	-	-	-	-	-
group_143		hypothetical protein	Pyrimidine-specific ribonucleoside hydrolase RihA	Decaprenyl diphosphate synthase	-	-	-	-	-	-	-	-	+	-	-	-	-	-	-	-
trpS2_2		Tryptophan-tRNA ligase 2			-	-	-	-	-	-	-	-	-	-	+	-	-	-	-	-
group_1540		ISL3 family transposase IS2001			-	-	-	-	-	-	-	-	-	-	+	-	-	-	-	-
group_1544		ISL3 family transposase IS2001			-	+	-	-	-	-	-	-	-	-	-	-	-	-	-	-
group_18		Long-chain-fatty-acid-CoA ligase FadD15			-	-	-	-	-	-	-	-	-	-	+	-	-	-	-	-
group_2		hypothetical protein	hypothetical protein	Oligo-1,6-glucosidase	-	-	-	-	-	-	+	-	-	-	-	-	-	-	-	-
lacZ_1		Beta-galactosidase			-	-	-	+	-	-	-	-	-	-	-	-	-	-	-	-
group_23		hypothetical protein	Phosphoglucomutase	hypothetical protein	-	-	-	-	-	-	-	-	+	-	-	-	-	-	-	-
group_25		hypothetical protein	Lactose permease	Beta-galactosidase	-	-	-	-	-	-	-	-	-	-	+	-	-	-	-	-
group_27		hypothetical protein	hypothetical protein	Leucine-tRNA ligase	-	-	-	+	-	-	-	-	-	-	-	-	-	-	-	-
cmk		Cytidylate kinase			-	-	-	-	-	-	-	-	-	-	+	-	-	-	-	-
alaS_1		Alanine-tRNA ligase			-	-	-	-	-	-	-	-	-	-	+	-	-	-	-	-
tetM		Tetracycline resistance protein TetM from transposon Tn916			-	-	-	-	-	-	-	-	-	-	+	-	-	-	-	-
group_37		hypothetical protein	Polyisoprenyl-teichoic acid-peptidoglycan teichoic acid transferase TagU	hypothetical protein	-	-	-	-	-	-	-	-	-	-	+	-	-	-	-	-
group_40		hypothetical protein	Polyisoprenyl-teichoic acid-peptidoglycan teichoic acid transferase TagU	Polyisoprenyl-teichoic acid-peptidoglycan teichoic acid transferase TagU	-	-	-	-	-	-	-	-	-	-	+	-	-	-	-	-
mfd_3		Transcription-repair-coupling factor			-	-	-	-	-	-	-	-	-	-	+	-	-	-	-	-
group_44		hypothetical protein	hypothetical protein	hypothetical protein	-	-	-	-	-	-	-	-	-	-	+	-	-	-	-	-
pbpD		Penicillin-binding protein 4			-	-	-	-	-	-	-	-	-	-	+	-	-	-	-	-
mntH_2		Divalent metal cation transporter MntH			-	-	-	-	+	-	-	-	-	-	-	-	-	-	-	-
group_50		hypothetical protein	Long-chain-fatty-acid-CoA ligase FadD15	dTTP/UTP pyrophosphatase	-	-	-	-	-	-	-	-	-	-	+	-	-	-	-	-
oxc_2		Oxalyl-CoA decarboxylase			-	-	-	-	-	-	-	-	+	-	-	-	-	-	-	-
group_54		hypothetical protein	hypothetical protein	hypothetical protein	-	-	-	-	-	-	-	-	-	-	+	-	-	-	-	-

Prokka annotation	*Gln*	Pfam annotation	*Mur*
SK-1	10 (Q2/H3/M2/P1/B1/D1)	SK-1	13 (5 glutamate ligase domain/6 middle domain/2 catalytic domain)
SK-2	10 (Q2/H3/M2/P1/B1/D1)	SK-2	13 (5 glutamate ligase domain/6 middle domain/2 catalytic domain)
SK-3	10 (Q2/H3/M2/P1/B1/D1)	SK-3	13 (5 glutamate ligase domain/6 middle domain/2 catalytic domain)
SK-4	9 (Q2/H/M2/P1/B1/D1)	SK-4	13 (5 glutamate ligase domain/6 middle domain/2 catalytic domain)
SK-5	10 (Q2/H3/M2/P1/B1/D1)	SK-5	13 (5 glutamate ligase domain/6 middle domain/2 catalytic domain)
SK-6	10 (Q2/H3/M2/P1/B1/D1)	SK-6	13 (5 glutamate ligase domain/6 middle domain/2 catalytic domain)
SK-7	10 (Q2/H3/M2/P1/B1/D1)	SK-7	13 (5 glutamate ligase domain/6 middle domain/2 catalytic domain)
SK-8	10 (Q2/H3/M2/P1/B1/D1)	SK-8	13 (5 glutamate ligase domain/6 middle domain/2 catalytic domain)
SK-9	10 (Q2/H3/M2/P1/B1/D1)	SK-9	13 (5 glutamate ligase domain/6 middle domain/2 catalytic domain)
SK-10	10 (Q2/H3/M2/P1/B1/D1)	SK-10	12 (4 glutamate ligase domain/6 middle domain/2 catalytic domain)
SK-11	10 (Q2/H3/M2/P1/B1/D1)	SK-11	13 (5 glutamate ligase domain/6 middle domain/2 catalytic domain)
SK-12	10 (Q2/H3/M2/P1/B1/D1)	SK-12	13 (5 glutamate ligase domain/6 middle domain/2 catalytic domain)
SK-13	10 (Q2/H3/M2/P1/B1/D1)	SK-13	13 (5 glutamate ligase domain/6 middle domain/2 catalytic domain)
SK-14	10 (Q2/H3/M2/P1/B1/D1)	SK-14	12 (4 glutamate ligase domain/6 middle domain/2 catalytic domain)
SK-15	10 (Q2/H3/M2/P1/B1/D1)	SK-15	13 (5 glutamate ligase domain/6 middle domain/2 catalytic domain)
SK-16	10 (Q2/H3/M2/P1/B1/D1)	SK-16	13 (5 glutamate ligase domain/6 middle domain/2 catalytic domain)

GlnQ	Glutamine transport ATP-binding protein
GlnH	ABC transporter glutamine-binding protein
GlnM	putative glutamine ABC transporter permease protein
GlnP	putative glutamine ABC transporter permease protein
GlnB	Nitrogen regulatory protein P-II
GlnD	Bifunctional uridylyltransferase/uridylyl-removing enzyme
